# Real-Time and Spatially Resolved Epigenetic Dynamics Tracking Beyond DNA Methylation via Live-Cell Epigenetic Sensors in 3D Systems

**DOI:** 10.3390/bios16040188

**Published:** 2026-03-25

**Authors:** Aqsa Tariq, Iram Naz, Fareeha Arshad, Raja Chinnappan, Tanveer Ahmad Mir, Mohammed Imran Khan, Ahmed Yaqinuddin

**Affiliations:** 1 College of Medicine, Alfaisal University, Takhassusi Road, P.O. Box 50927, Riyadh 11533, Saudi Arabia; atariq@alfaisal.edu (A.T.); inaz@alfaisal.edu (I.N.); farshad@alfaisal.edu (F.A.); rchinnappan@alfaisal.edu (R.C.); 2 King Faisal Specialist Hospital and Research Center, Takhassusi Street, P.O. Box 3354, Riyadh 11211, Saudi Arabia; tmir@kfshrc.edu.sa; 3 King Faisal Specialist Hospital and Research Center, Al Rawdah Street, P.O. Box 40047, Jeddah 23422, Saudi Arabia; mikhan@kfshrc.edu.sa

**Keywords:** non-methyl epigenetic modifications, histone PTMS, epigenetic sensors, genetically encoded sensors, CRISPR/dCas-based sensors: live-MIEL

## Abstract

**Background**: Gene expression and cellular identity are regulated by epigenetics that occurs through chromatin modifications, RNA changes, chromatin accessibility, and three-dimensional genome organization. Although DNA methylation has been the focus of most epigenetics studies in the past, other non-methyl epigenetic processes, including histone post-translational modifications (PTMs), epitranscriptomic marks, and chromatin remodeling, are dynamic, reversible, and context-dependent, and thus are difficult to accurately interrogate using endpoint sequencing-based assays, especially in heterogeneous tissues, developing systems, and therapeutic response environments. **Scope and Approach**: The present review discusses epigenetic modifications other than DNA methylation regarding sensor-based technologies that can measure live, dynamic, and spatially resolved measurements. Epigenetic sensors include any genetically encoded sensors (GECs) based on resonance energy transfer, CRISPR/dCas-derived sensors, or aptamer-based sensors, and hybrid biochemical/imaging sensors that can be used in live or semi-live settings. It lays emphasis on the technologies, which have been developed recently, that allow real-time kinetic measurements, working in three-dimensional and organoid models, and being applied to disease-relevant perturbations. On these platforms, performance properties such as specificity, sensitivity, spatial and temporal resolution, ability to perform dynamic versus locus-specific interrogation, and perturbed endogenous chromatin states are compared. **Key Conclusions and Outlook**: Together, these sensing strategies are complementary to the traditional methods of measuring epigenomics in that they show epigenetic dynamics unobservable with static measurements. We list the important technical issues, including specificity, quantitation, multiplexing, and chromatin perturbation, and report the barriers and solutions in development and design. Lastly, we provide a conceptual map of how live epigenetic sensing and multi-omics and translational models can be integrated, and how the two methodologies can be used to develop functional epigenetics and guide disease modeling and drug development.

## 1. Introduction

A key role in the diverse biological processes from development to disease pathogenesis is played by epigenetic modifications, which are context-dependent and reversible structural and chemical changes in chromatin and RNA that regulate gene expression without changes in the core DNA sequence [1]. These modifications, which include DNA methylation, histone modifications, nucleosome positioning, non-coding RNAs, and chromatin accessibility, collectively form a complex regulatory network that dictates gene expression and cellular phenotype [2]. The dysregulation of epigenetic machinery contributes to the initiation and progression of disease-related gene expression programs using specific molecular pathways, including aberrant histone acetylation and DNA methylation in cancer activation of oncogenes and silencing of tumor suppressors, altered chromatin accessibility and histone modification in neuronal dysfunction mechanisms in neurodegenerative disease, and age-associated chromatin reorganization and epigenetic drift that affect metabolic regulation and cellular survival [3].

Historically, DNA methylation (5-methylcytosine and related derivatives) has been a primary focus of epigenetic research because the marks are relatively stable, abundant, and responsive to mapping. An emerging need exists to investigate other crucial epigenetic markers to fully comprehend the role of the epigenome in health and disease [4]. Among these, histone PTMs like acetylation, ubiquitination, methylation, and phosphorylation exert powerful control and employ effector proteins (“readers”) and engage in crosstalk, giving a combinative “histone code” with major effects in cancer, developmental biology, and aging [5]. Progressively, the need is being identified to go beyond DNA methylation to capture these additional layers, especially in their temporal, spatial, and functional dynamics in live systems.

Conventional approaches for epigenetic modification analysis, including bisulfite sequencing of DNA methylation, chromatin immunoprecipitation (ChIP) or CUT&RUN sequencing of histone marks, and assay of transposase-accessible chromatin sequencing (ATAC-seq) of chromatin accessibility, have given invaluable insights into the epigenetic landscapes. Still, they tend to mask the natural heterogeneity of various cell types and do not provide the spatiotemporal resolution of dynamic epigenetic variations in live cells and in complex 3D systems [6,7]. Additionally, these techniques are typically endpoint assays requiring cell fixation or lysis.

This is where sensor-based, live-cell approaches are crucial for translation into inherently dynamic and heterogeneous diseases, for example, cancer or neurodegeneration [8]. For example, tumor microenvironments drive spatially distinct epigenetic states, and the drug response often involves rapid epigenetic remodeling. Live sensors permit kinetic tracking of these changes in 3D models, for example, organoids, describing the cell-to-cell interactions, spatial context, and elements that endpoint assays oversee.

This review goes beyond the classical focus on DNA methylation to discuss epigenetic changes through sensor-based technologies. In contrast to previous reviews that mainly summarize epigenomic profiling technologies or static imaging-based technology, we are particularly interested in live and dynamic sensing of epigenetics. Special focus is placed on sensor measurements in disease-relevant and three-dimensional cell cultures, like organoids and microenvironment-controlled cultures, in which the epigenetic kinetics can be different from those of two-dimensional cultures.

The term “epigenetic sensors” includes GECs, Förster Resonance Energy Transfer (FRET) based biosensors, CRISPR/dCas-derived probes, aptamers, intrabodies (e.g., Modification-specific intracellular antibodies “mintbodies”), Fab fragments (antigen-binding fragments), proximity-based systems, and hybrid biochemical-imaging systems that can be used with live or semi-live measurements. Among these modalities, we compare sensor capabilities and limitations based on sensitivity, specificity, spatial and temporal resolution, perturbation of endogenous chromatin, and the sensor applicability to dynamic or three-dimensional states. Lastly, we highlight future applications in cancer, aging, and therapeutic response monitoring, and summarize the critical technical and conceptual challenges to be overcome to support physiologically realistic and translationally relevant readings in epigenetic measurements.

## 2. Epigenetics Beyond DNA Methylation

**Figure 1:**
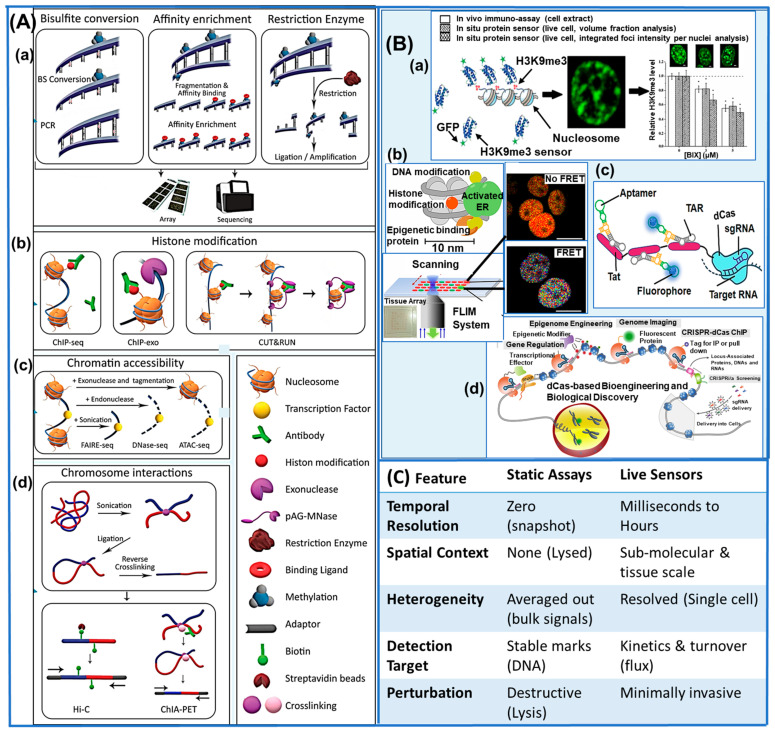
Comparison between (**A**) traditional epigenomic assays (**a**) DNA methylation assessment methods via bisulfite conversion-based, affinity enrichment-based, and restriction enzyme-based techniques, (**b**) histone modifications detection (ChIP-seq, ChIP-exo, CUT&RUN), (**c**) chromatin accessibility analysis (FAIRE-seq, DNase-seq, ATAC-seq), (**d**) chromatin interaction (Hi-C, ChIA-PET) (under CC-BY 4.0 license [9]), and (**B**) emerging live imaging epigenetic approaches (**a**) recombinant protein sensors (under CC license [10]), (**b**) FLIM-FRET sensor (under CC-BY 4.0 license [11]), (**c**) RNA-aptamer fluorescent sensors (under CC-BY 4.0 license [12]), (**d**) dCas-based epigenetic sensing (under CC-BY 4.0 license [13]) and (**C**) summarized comparison.

Figure 1A presents the summary of all the epigenetic modifications and their traditional detection methods.

### 2.1. Histone Modifications

The epigenetic marks referred to as histone PTMs are possibly one of the most functionally diverse. Histone N-terminal tails and histone variants are subject to a variety of PTMs, like lysine acetylation and methylation, ubiquitination, phosphorylation, and other acylations, that can be combined to control chromatin structure and gene expression. Histone modification refers to the addition of chemical groups to the histone proteins [14].

The various functions of chromatin, like compaction, transcription, DNA repair, and genome stability, are mediated by these histone modifications. Histone PTMs are controlled by “writers” that add marks (HATs and HMTs), “erasers” that remove marks (histone demethylases (HDMS), histone deacetylases (HDACs)), and “readers” (PHD fingers, bromodomains, chromodomains) that selectively bind modified residues to interpret epigenetic signals [14].

Acetylation usually happens on the lysine residues located on the histone tails, e.g., H3K9, H3K14, etc. The lysine residues (positively charged basic groups) are neutralized by acetylation. Thus, the interaction between histones and DNA also becomes weak. Therefore, these factors shape the chromatin structure. Acetylation prevents chromatin from compacting and depends on whether metabolism is occurring or not. Inside the cell, transcription is promoted.

Histone methylation is a highly context-dependent signal, considering that mono-, di-, or tri-methylation on specific lysines or arginines can either activate or repress transcription. For example, whereas H3K4me3 forms active promoters, H3K27me3 and H3K9me3 are specific marks of transcriptional repression and heterochromatin. These methylation states are generally reversible and context-dependent, and their dynamics should not be monitored as static marks [15].

Monoubiquitylation of H2A and H2B histones plays several essential roles in the regulation of transcription, response to DNA damage, and chromatin remodeling [16]. Although relatively low in stoichiometry, this modification can have significant regulatory consequences through directing other histone marks, underscoring the interplay of epigenetics.

Histone phosphorylation often serves as a quick-signaling system, coupling extracellular signals or cell-cycle events with the changes in chromatin. For example, the phosphorylation of H3S10 is associated with mitosis and transcriptional activation in response to stress signals [17].

Apart from chemical modifications, histone variants including H2A.Z and H3.3 change nucleosome stability and positioning, thereby contributing to transcriptional memory, enhancer function, and genome integrity. Contrary to recognized histones, the deposition of variants occurs in a DNA replication-independent manner and represents an additional dynamic layer of chromatin regulation [18]. Combined, histone PTMs and variants constitute a combinatorial and highly dynamic “histone code” that cannot be adequately comprehended by static endpoint measurements alone.

### 2.2. RNA Modifications

RNA modifications, a crucial epigenetic regulation, connect the chromatin state to post-transcriptional gene control. Of these, N6-methyladenosine (m6A) represents the most abundant internal modification in eukaryotic mRNA and influences RNA stability, splicing, nuclear export, and translation efficiency [19]. The m6A landscape is dynamically regulated by writer, eraser, and reader proteins, enabling rapid adaptation to developmental cues, stress, and disease states.

Other RNA modifications, including m1A, pseudo-uridylation (Ψ), and 5-hydroxymethylcytosine (5hmC) on RNA, continue to increase the transcriptome regulatory potential [20]. Each of these modifications can influence RNA structure, ribosome engagement, and protein synthesis fidelity. More importantly, RNA modifications often change more quickly against chromatin marks, placing them as the critical mediators of short-term cellular adaptation [21].

Most of the recent approaches depend on immunoprecipitation or chemical conversion followed by sequencing; thus, they provide population-averaged snapshots that mask temporal fluctuations and cell-to-cell heterogeneity [22].

### 2.3. The Chromatin Accessibility

The availability of chromatin corresponds to the physical readiness of DNA to interact with transcription factors, polymerases, and regulatory complexes. ATAC-seq methods made it possible to map accessible regions of chromatin, leading to the determination of active promoters and enhancers [6]. Although single-cell and combinatorial indexing ATAC-seq techniques have greatly increased both cellular resolution and scalability, cell lysis and fixation remain required by these methods, thus only offering static measurements of accessibility under conditions of no motion and spatial resolution of accessibility variations in living systems.

In cells, chromatin accessibility is a dynamic process that keeps changing due to events occurring in transcriptional bursting, the cell cycle, differentiation, and stress responses. This dynamic nature is particularly important in a diseased state, such as cancer, in which different cells within a tissue have various levels of dynamic chromatin accessibility [23].

There are multiple models created using the principles of accessibility maps [24]. Besides linear accessibility, chromatin is further organized at the hierarchy level by euchromatin and heterochromatin domains. This suggests that the three-dimensionality of chromatin, among others, is essential.

### 2.4. Integration of Multi-Omics in Epigenetic Crosstalk

Epigenetic regulation is controlled by highly coordinated and combinatoric interactions of many molecular layers, unlike independent marks. The histone modifications that have been identified to work together with DNA methylation in setting up permissive or repressive chromatin states, chromatin accessibility regulates transcription factor binding, and RNA modifications regulate chromatin by the RNA-binding proteins and chromatin-associated RNAs [25]. In Drosophila and mammals, RNA modifications like m6A play a key role in the transcription initiation regulation and elongation by regulating chromatin-related RNA processing and factor recruitment, which have mechanistic connections between RNA modification and chromatin regulation [26].

Multi-omics systems that combine complementary epigenetic and transcriptional layers have been of growing importance to measure such crosstalk. As an illustration, now combined CUT&Tag or CUT&RUN profiling of histone changes in conjunction with bulk or single-cell RNA sequencing has made it possible to directly associate changes in chromatin state with transcriptional output. In contrast, paired single-cell ATAC-seq and scRNA-seq systems have made coordinated changes in chromatin accessibility and gene expression during differentiation and disease progression [27]. Even more recently, spatial multi-omics approaches have started to encode chromatin features and transcriptomes in intact tissue environments, revealing spatially confined epigenetic programs that cannot be discovered in dissociative assays.

Although these developments exist, the majority of multi-omics studies are intrinsically correlative or inferential, with the data of various modalities being usually obtained on different cells or time periods and subject to computational combination. This creates issues of time mismatch, technical bias in a modality, and the fact that they rely on statistical inference and not on actual mechanistic validation [28]. Although perturbation-coupled experiments and joint profiling systems have started to overcome such constraints, real-time and causal interactions between epigenetic changes across layers remain challenging to measure. Accordingly, the existing multi-omics systems offer robust associative data but are not able to directly monitor dynamic epigenetic crosstalk as it occurs in biological systems.

### 2.5. 3D Genome Architecture

The genome is divided into organizational levels in the nucleus in the form of topologically associating domains and compartments. There are contacts between enhancers and promoters, insulated domains, and long-range repression, all of which are made possible by 3D genome structure [29].

Chromosome conformation capture approaches, for example, Hi-C, have successfully decoded such interactions on the genome scale by identifying common principles. However, the Hi-C approaches require fixing and population-based analyses that hamper their effectiveness in measuring the dynamics of such arrangements or unusual cell configurations [30].

Live-cell imaging methods have clarified that genome structure is much more dynamic than has been previously realized [31]. Chromatin loops can emerge and disassemble on time scales that are relevant to transcription regulation, and this is tightly coupled to histone modifications and chromatin accessibility. A visualization method that simultaneously offers both spatial and temporal information is required to observe the role of epigenetic marks within and their impact on the 3D structure of the genome.

## 3. Live Cell and Dynamic Sensors for Epigenetic Modifications

Table 1 provides a summary of some of the common live-cell epigenetic sensors.

### 3.1. Genetically Encoded Sensors

#### 3.1.1. Mintbodies

In mintbodies, single-chain variable fragments (scFv) of modification-specific antibodies are fused to fluorescent proteins. Unlike conventional antibodies, mintbodies are sufficiently small and stable to withstand the reducing cellular environment and diffuse freely in the nucleus. For example, Chung et al. constructed a FRET probe using yellow fluorescent protein bound to the N-terminus of an acetyl H3K9-specific scFv secured to a cyan fluorescent protein to quantify spatial and temporal dynamic changes in histone acetylation [42]. The FRET efficiency and fluorescence intensity in the nucleus were both increased following histone-deacetylase inhibitor treatment when the FRET probe was expressed in human cells.

A characteristic example is the H3K9ac mintbody, an indicator of actively transcribing chromatin found enriched at promoters and enhancers [43]. Instead of an immobile focus, the mintbody shows a dynamic shuttling between the cytosol and the nucleus according to the acetylation state. Inhibition of HDACs by pharmacological inhibitors causes a quick rise in nuclear levels, suitable for observing the dynamic state of histone acetylation in real time.

Notably, mintbodies have been used successfully in vivo. The H3K9ac mintbodies in vivo showed the spatiotemporal dynamics of chromatin activation events during embryonic development, including the epigenetic reprogramming phase during the maternal-zygotic transition [43]. Similarly, a study developed an inducible fluorescent mintbody to observe spatiotemporal dynamics of H3K27 trimethylation and its interaction with TopoIIα [44].

To stabilize the most scFvs in live cells, the Frankenbodies production technology considered using the scaffold engineering approach. This is where the CDRs are transferred to stable scFv scaffolds, known to have optimal intracellular folding [45].

**Figure 2:**
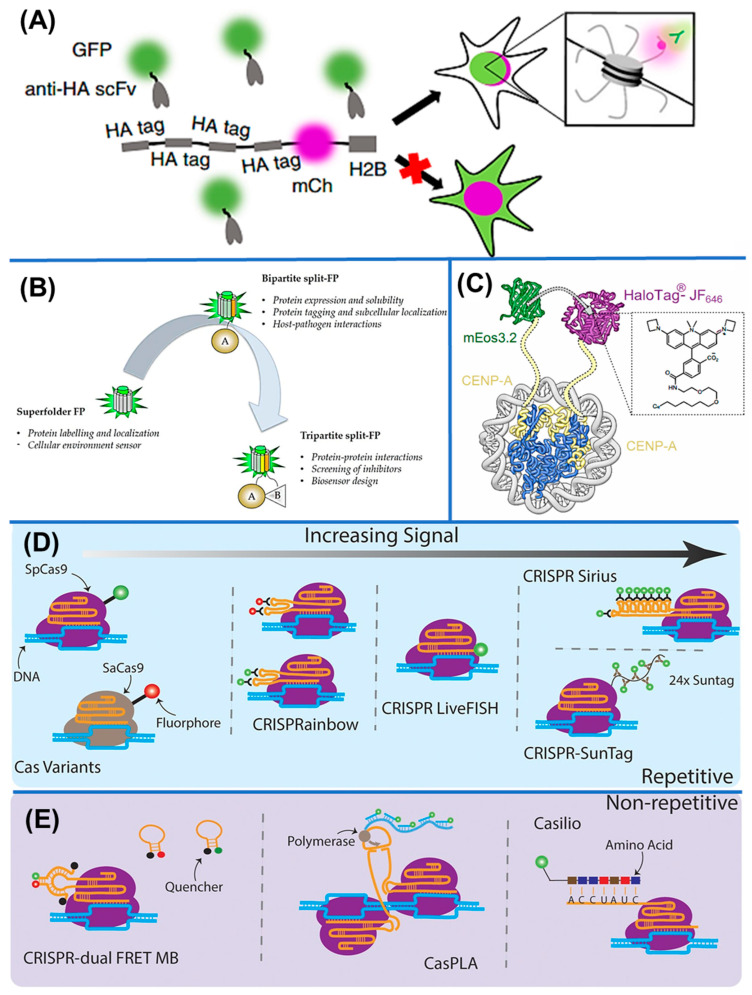
(**A**) HA-Frankenbodies (five chimeric anti-HA scFvs in living U2OS cells. Each scFv fused to GFP (green) was co-expressed with HA-tagged histone H2B fused to mCherry (magenta). Nuclear co-localization indicates successful binding, while the red cross marks absence of binding (under CC-BY 4.0 license [45]), (**B**) superfolder and split fluorescent protein detection systems (under CC-BY 4.0 license [46]), (**C**) FRET pairs tagging CENP-A with mEos3.2 or Halotag JF646 protein (under CC-BY 4.0 license [35]), (**D**) CRISPR-based live-cell imaging technologies targeting repetitive genomic sequence (under CC-BY 4.0 license [47]), and (**E**) CRISPR-based live-cell imaging technologies for non-repetitive loci detection [47].

Frankenbodies have made possible the imaging of histone variants and chromatin proteins tagged at the single-molecule level in real-time. Zhao et al. designed an HA frankenbody to track single HA-tagged histones in U20S cells and single mRNA translation dynamics (Figure 2A). With HA-tag frankenbodies, the following has been found: nucleosomes are not uniformly distributed throughout the chromatin but form chromatin clutches, a nanoscale chromatin structure that challenged the conventional representation of chromatin as a static chromatin fiber [45].

#### 3.1.2. Frankenbodies

In another study, Zhao et al. have engineered a new HA frankenbody that lights up in multiple colors, HA-tagged nuclear, cytoplasmic, and membrane proteins in various types of living cells [45]. They tracked single mRNA translation dynamics in U2OS cells and neurons, two mRNA species in combination with SunTag, and HA-tagged protein expression in developing zebrafish embryos. Aside from epigenetic state sensing, frankenbodies have also been engineered for the detection of emerging protein translation and protein-protein interaction, emphasizing the versatility of the tool.

#### 3.1.3. Live-MIEL

Live cell microscopic imaging of epigenetic landscapes (Live-MIEL) differs from standard intensity-based sensing in that it focuses on the spatial distribution of chromatin. Live-MIEL analyzes the texture, distribution, and nuclear organization, as well as intensity, by using fluorescent tags on reader domains, which are highly specific for histone marks H3K9me3, H3K9ac, and H3K4me1. A study developed a Live-MIEL system with red fluorescent sensors that identify H3K9me3, H3K9ac, and H3K4me1 on live cells [48].

Such a method can detect fine changes in chromatin conformation that are not detectable by cell-scale assays or by imaging based solely on signal intensity. For example, modification-recognition probes genetically encoded have been used to monitor global remodeling of histone methylation landscapes in induced pluripotent stem cell (iPSC) differentiation [33]. Live-MIEL has been employed for differentiation stage separation, tumor cell type identification by phenotypes, and for the identification of drug-induced changes in chromatin, even when the level of global modification is unchanged [49].

Affinity tuning of the recognition probe is another major design concept in epigenetic sensing systems of live cells. High-affinity probes can bind modified histones strongly but compete with endogenous chromatin reader proteins, which can disrupt chromatin dynamics. Conversely, low-affinity probes have decreased signal sensitivity and detection accuracy as they might fail to detect modification effectively [50]. Thus, to achieve detection sensitivity with minimum interference with endogenous chromatin regulation, probe affinity should be carefully optimized.

The epigenetics is inferred by analyzing patterns of nuclear staining with machine learning in Live-MIEL imaging-based platforms. But Live-MIEL may be restricted in terms of its broader applicability as it depends on large training sets. Models, which have been trained on a particular cell type or experimental setting, might not be reliable for use on a formerly unobserved biological situation; this can reduce prediction accuracy and generalization [51].

### 3.2. RNA- and Aptamer-Based Sensors

Aptamers are structurally compact nucleic acid sequences, selected for high specificity. One reason aptamers have been considered for epigenetics is their suitability for sensing histone modifications and RNA epigenetics, and complementing current sensing methods. The concept of aptamers has been further extended with the use of fluorescent dyes or the CRISPR system for epitranscriptome modification analysis [52]. They can be used for m6A modifications analysis. Zhang et al. reported single-cell dynamics in viral infection monitored in real-time with an RNA methylation sensor (ms6A) [53].

Lately, the fluorogenic RNA aptamers, for example, Pepper, have permitted the construction of RNA-based sensors that do not require RNA G-quadruplex motifs. For instance, Pepper-based sensors have been used to measure intracellular levels of S-adenosylmethionine (SAM) in living cells, to enable spatiotemporal visualization of SAM biosynthesis and methionine adenosyltransferase activity, thereby explaining metabolite-dependent regulation of epigenetic methylation [54]. Compared to protein sensors, aptamers have the advantages of modularity and ease of synthesis, but still face challenges regarding their stability within cells and delivery.

### 3.3. Resonance Energy Transfer-Based Sensors

#### 3.3.1. FRET-Based Sensors

FRET sensors are designed to translate these modifications into conformational changes that are detectable as a shift in the energy transfer efficiency between fluorescent proteins. Figure 2B presents a few common types of fluorescent proteins (FP), including superfolder FP, bipartite split-FP, and tripartite split-FP. Most designs feature a histone tail peptide, a reader domain specific to the relevant modification, and flanking donor-acceptor fluorophores, which means intramolecular binding upon modification changes the distance between the fluorophores and produces a measurable FRET signal [55].

FRET sensors have been developed for a variety of histone PTMs, including but not limited to H3S28 phosphorylation, H4K12 acetylation, and other repressive methylation marks, including H3K9me3 and H3K27me3. The investigation of these methods to monitor H3K9me3 and H3S10p changes during mitosis provided an anticorrelation relationship between these marks and showed coordinated chromatin remodeling during cell division [32].

A study reported an H3K27me3 FRET sensor in living cancer cells with an apoptotic marking coupling option [56]. Furthermore, a MOF histone acetyltransferase activity biosensor with a genetically encoded FRET reporter in vitro and live cells has been developed [57]. Researchers have gained much better SMT by designing novel FRET pairs based on the photo-modulatable donor fluorophores (mEos3.2 or PA-JF549) with a photostable acceptor dye, in live mammalian cells and chromatin binding proteins (Figure 2C) [35]. Application of these sensors has been instrumental in dissecting the coupling between signaling pathways, chromatin modification, and transcriptional activation in the context of stress responses and cell-cycle progression.

#### 3.3.2. FLIM-FRET for Quantitative Imaging

Intensity-based FRET measurements are susceptible to several factors, such as fluorophore concentration, photobleaching, and light scattering. Instead, FLIM (Fluorescence lifetime imaging)-based FRET overcomes these limitations in measuring changes in the donor fluorescence lifetime, which is an accurate reflection of energy transfer efficiency.

FLIM-FRET, thus, allows the quantitative, calibration-free detection of epigenetic changes, with a particular advantage in thick tissues and 3D cultures. The phasor-based analysis of FLIM further discriminates true FRET signals from autofluorescence, enhancing robustness in complex biological samples. For instance, Liu et al. developed FLIM-FRET to study epigenetic biomarkers within single-nucleosome proximity of estrogen receptor-dependent genes in breast cancer samples (Figure 1B(b)). They screened 11 epigenetic marks, including histone modifications, oxidative forms of DNA methylation, and methyl-binding domain proteins, which resulted in the identification of H4K12ac and H3K27ac as possible epigenetic therapeutic targets [11]. Another study reported FLIM-FRET-based chromatin sensors with histone pairs (H2B-eGFP/H2B-mCherry) and phasor image-correlation spectroscopy for the quantitative mapping of nucleosome-level chromatin compaction in live cells. This study revealed that ATM- and RNF8-stimulated chromatin decompaction and surrounding compact chromatin foci dynamically regulate the spatial organization of repair sites at double-strand breaks during the DNA damage response [58].

#### 3.3.3. Multiplexed Dark FRET (MDF)

Because the multiplexing of multiple FRET sensors is severely limited by spectral overlap, the MDF strategy circumvents this problem by employing nonfluorescent (“dark”) acceptors, enabling multiple donor fluorophores to be observed in one shot through changes in lifetime [59]. This permits the parallel sensing of many epigenetic events in the same cell, a critical requirement for epigenetic crosstalk in 3D systems. For example, the dark yellow fluorescent protein ShadowY is an optimized FLIM-FRET acceptor with a very low quantum yield and better folding behavior that can be used to detect conformational changes and protein interactions in living cells in a highly sensitive manner when directly coupled with one of the many fluorescent donors, mEGFP or Clover variants [60]. These systems of dark acceptors reduce spectral crosstalk and are the basis of a new generation of MDF systems that strive to monitor several intracellular signaling or regulatory events simultaneously.

### 3.4. CRISPR/dCas-Based Sensors

Catalytically inactive Cas9-one factor, dCas9, fused with fluorescent proteins, enables programmable visualization of specific genomic loci in living cells. By designing guide RNAs to target repetitive or unique sequences, researchers can track chromatin motion, locus repositioning, and long-range interactions in real time. Initial methods used sequence-specific sgRNAs to target fluorescently tagged dCas9 proteins to label repetitive regions in the genome, as depicted in Figure 2D [47]. For example, SgRNA-aptamer has been designed for genome loci imaging through CRISPR-dCas9 in mouse ES cells [37]. Similarly, Chen et al. used SgRNA and EGFP-tagged endonuclease-deficient Cas9 protein for live cell imaging of repetitive regions in telomerase and coding genes [61].

CRISPRainbow extends this using RNA aptamers that recruit multiple fluorescent proteins for simultaneous tracking of several genomic regions [62]. For example, bacteriophage-derived sgRNA scaffolds (CRISPRainbow) using RNA stem loops (MS2, PP7, and BoxB) were integrated into sgRNA scaffolds and allowed binding fluorescent coat proteins [63]. Multicolor CRISPR labelling methods can be used to visualize a large number of genomic loci at once, to measure intranuclear distances, and to study the dynamics of chromatin compaction in living cells [64].

Later, the signal-to-noise ratio and multiplexing using engineered sgRNA scaffolds and signal amplification plans were enhanced. Subsequent versions, such as CRISPR-Sirius and CRISPR-SunTag, enhanced levels of fluorescence by using more elaborate RNA stem loops or recruitment of a number of fluorescent proteins and could detect low-copy sequences or weakly repeated sequences [47]. Other methods like CRISPR LiveFISH involve the use of dye-labeled sgRNAs to visualize the targets of the genome [65].

Other platforms have been designed to work with non-repetitive genomic regions (Figure 2E), such as Casilio, which uses RNA-binding proteins to amplify signal through recruiting proteins and CRISPR-dual FRET molecular beacon systems, which release fluorescence only when the target is bound [47]. Other technologies like CasPLA enable one to detect nucleotide variations at the single-nucleotide level using proximity-based signal amplification [66]. In addition to DNA-targeting CRISPR systems, the programmable sensing of the epitranscriptome has been expanded by RNA-targeting CRISPR systems. The technologies based on Cas13 allow direct identification of the RNA molecules and modifications.

SHERLOCK exploits Cas13’s collateral cleavage activity to amplify the detection of RNA modifications with extraordinary sensitivity [67]. All these CRISPR-based imaging technologies allow a high-resolution multiplexed visualization of genomic organization and epigenetic regulation in live cells.

### 3.5. Super-Resolution Visualization

Single-molecule localization microscopy (STORM, PALM) and stimulated emission depletion (STED) microscopy have resolved the epigenetic organization at nanometer resolution. These methods disclosed that chromatin is partitioned into discrete clutches of nucleosomes whose spatial architecture differs between active and repressive domains [68]. Furthermore, live-cell STED imaging revealed fast chromatin “breathing” and reader protein diffusion, offering unparalleled insight into epigenetic dynamics at physiological timescales. For instance, STED and stimulated emission double depletion (STEDD) imaging with silicon-rhodamine-Hoechst DNA labeling has been used to resolve high-resolution images of the three-dimensional distribution of nuclear DNA in cells of zebrafish embryos, and the chromatin architecture can be observed at depths of several tens of micrometers [69]. These types of super-resolved imaging are solid platforms on which to study spatial genome organization and chromatin structural dynamics that relate to epigenetic regulation in living tissues.

### 3.6. Nanomaterial-Based Sensors

Nanotechnology offers the benefits of signal amplification, surface modification, and miniaturization that are crucial for highly sensitive epigenetic detection. Electrochemical biosensors that are functionalized using histone peptides or reader domains offer a means of translating epigenetic information into an electrical response [70]. Graphene, carbon nanotubes, and metal nanoparticles have improved the sensitivity to the femtomolar level for histone-modifying enzyme activity detection [71]. LSPR-based sensors have the capability for label-free analysis of histone modifications based on a change in the refractive index. These have potential applications in point-of-care epigenetics. For instance, heterochromatin-associated histone marks, including H3K9me3 and H3K27me3, have been imaged with plasmonic nanoparticle probes with hyperspectral colorimetric imaging to produce quantifiable spectral changes that are sensitive to the spatial arrangement of these epigenetic modifications. In this study, distance-dependent plasmonic coupling generated measurable spectral shifts that reflected the spatial organization of these epigenetic markers [72]. This method is a nanoprobe-based high-spatial mapping of histone modification reorganization, including the repositioning of repressive chromatin markers during oncogene-induced senescence, which can be used to explore the dynamics of chromatin reorganization in disease progression.

### 3.7. Microfluidic Platforms

Microfluidic and nanofluidic supports the precise regulation and cellular microenvironment control, including gradients for nutrients, mechanical stress, and drugs. For example, a functionalized asymmetric nanochannel device with monotriazole-containing p-sulfonatocalixarene probes has been constructed to selectively determine the presence of lysine methylated peptides and distinguish between the various states of methylations by converting the molecular recognition event into measurable ionic current variations, allowing real-time measurements of the methyltransferase activity at sub-picomole sensitivity [73]. Epigenetic sensors incorporated into microfluidics facilitate the real-time chromatin dynamics tracking in physiologic settings. This is particularly important for analysis of tumor heterogeneity, drug resistance, and plasticity for the epigenetic state in 3D cultures and organoids [74].

### 3.8. Optogenetic Control and Readouts

Optogenetics brings spatiotemporal precision to epigenetic analysis: light-controllable recruitment of epigenetic writers or erasers enables chromatin manipulation with precision, with simultaneous sensing probes capable of detecting the respective epigenetic dynamics [75]. There are also optogenetic methods, which aim at reducing sensor interference with endogenous chromatin readers. As an example, a light-activated modification sensor (MPP8-LAMS) was designed to image H3K9me3 histone methylation in living cells, where blue-light stimulation causes the nucleus translocation of the sensor by an AsLOV2 domain and, thereby, allows on-demand detection of heterochromatin loci while reducing prolonged chromatin-binding and resulting in heterologous perturbation of natural epigenetic regulation [76]. A closed-loop approach thus provides a means to analyze epigenetic regulation and feedback in living cells causally.

### 3.9. Limitations of Dynamic Sensors

Irrespective of the sensing modality used, the epigenetic sensor design is controlled by basic trade-offs in spatial resolution, temporal resolution, genomic coverage, throughput, and perturbation of native chromatin state. As shown in Table 2, to obtain high genomic or molecular specificity, such high specificity is frequently traded off against compatibility with live-cell imaging. In contrast, dynamic sensing often requires reduced locus coverage or simplified molecular readouts.

Another and more significant factor in a large range of sensor types, including mintbodies, reader-domain fusion probes, FRET-based constructs, and dCas-based systems, is that they may interfere with endogenous chromatin regulation. Owing to the binding nature of these sensors to modified histones, DNA, or RNA, they naturally compete with natural chromatin readers on the same molecular target. This can be a stoichiometric competition that alters the effective occupancy of endogenous reader proteins and can have an effect on transcriptional outcomes. Additionally, extended binding or high expression of sensor constructs can change residence times of chromatin-binding factors or artificial chromatin decompaction or compaction, especially in heterochromatic segments.

Moreover, fluctuating imaging conditions will also cause artifacts, and a delicate balance using signal-to-noise ratios versus phototoxicity is needed such that the tracking remains truly non-perturbative [77]. In drug response biosensors, FRET-based sensors can be modified with modification-binding modules to detect intramolecular conformational changes as a consequence of modification [78]. The sensors have an upper hand over the traditional methods because they allow constant monitoring without interfering with cell division, development, or differentiation. For instance, recent FRET-based reporters have been utilized effectively to observe the dynamic kinetics of histone acetylation under conditions of HDAC inhibitor (Vorinostat) treatment, giving a real-time measure of drug effects at the single-cell level [79]. Likewise, enhanced sensors of DNA methyltransferase (DNMT) activity, based on the use of fluorescently-tagged cofactors or cationic conjugated polymers, have elucidated the immediate epigenetic changes and dynamics caused by inhibitors such as 5-azacytidine [80]. These methods have shown that epigenetic reactions to therapeutics are extremely varied within cell populations [34]. Different sources of this heterogeneity may include stochastic changes in chromatin state, cell-cycle dependent effects, and perhaps stable epigenetic subpopulations with other regulatory programs [77]. As an example, dynamic epigenetic sensing is clinically relevant through real-time monitoring of histone methylation and DNA methylation in cancer cells, in which particular epigenetic subpopulations show different sensitivities to drugs.

Such perturbation is different in sensor platforms. As an illustration, dCas9-based sensors offer locus-specific measurements of epigenetic states at the cost of sterically interfering with transcriptional machinery or chromatin rearrangements at specific locations when numerous guide RNAs are used [37]. Conversely, FRET-based histone sensors provide fast and reversible readouts of global modification dynamics with minor genomic impairment, but have no locus specificity. Several methods allow visualization of endogenous changes with relatively low molecular weight, e.g., mintbodies and intrabodies, although their affinity will have to be optimally adjusted to not perturb chromatin [36]. Sensor calibration, orthogonal validation, and controlled expression level are essential to overcome these limitations.

The sensing technology in epigenetics has, therefore, transformed the static biochemical tests into dynamic, programmable, and biologically contextual apparatus. Fusion of molecular recognition domains with optical, electrical, or nanophotonic transducers, coupled with microenvironmental control on microfluidics or organoid platforms, has provided the first opportunity to access epigenetic regulation in living systems [81]. To make sure that dynamic measurements precisely imitate the native epigenetic states, the convergent combination of low-perturbation sensor designs, multiplexed readouts, and quantitative authorization frameworks is the way forward.

## 4. Applications In Vivo/3D Culture/Organoid

**Figure 3:**
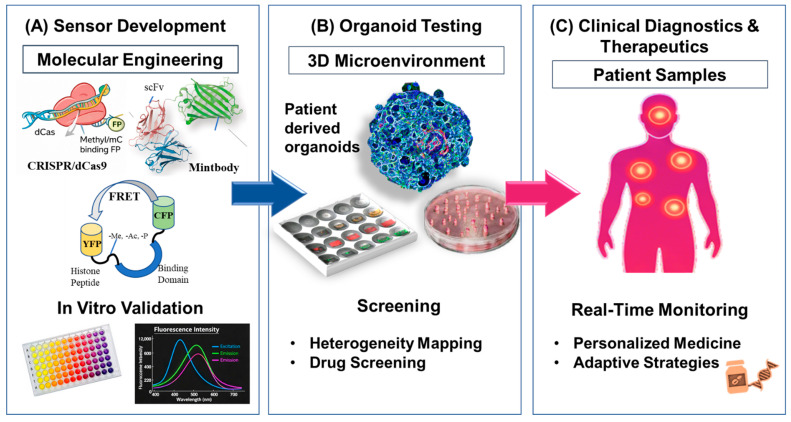
Schematic illustration of integration of epigenetic biosensors into organoid platforms, (**A**) sensor development using molecular probes, (**B**) testing in 3D microenvironment, and (**C**) employment on real samples.

Live-cell epigenetic sensors have extended epigenetic studies beyond mono-culture to a 3D, organoid, and in vivo ecosystem where tissue architecture, mechanical forces, and cell-cell interactions influence epigenetic regulation. Although these models are associated with significantly greater physiological relevance than 2D cultures, they present new imaging depth issues, sensor delivery, and interpretation of spatially heterogeneous signaling problems. In perfusion-based organ-on-chip models, there are early studies that demonstrate that epigenetic sensors are capable of measuring chromatin responses to biomechanical stimuli like shear stress in real time [82]. But its use in immune tumor co-cultures and stromal systems is still in its infancy. The integration of epigenetic biosensors into organoid platforms is shown in Figure 3.

Recent advances in 3D co-culture models have been mostly metabolomic and morphologic readout, but chromatin dynamics are underexplored. Live epigenetic imaging is a promising future in understanding mechanisms to elicit immune infiltration, stromal communication, and microenvironmental gradients to remodel chromatin states in vivo [83]. Besides live-cell epigenetic sensing technologies, several complementary spatial epigenomic profiling technologies have been utilized in three-dimensional culture systems and organoid models to describe chromatin states, epigenetic heterogeneity, and therapeutic response. Table 3 highlights some of the possible applications of epigenetic sensors and spatial epigenomic profiling approaches in 3D and in vivo.

### 4.1. 3D Cultures Complexity

There is a lack of optical penetration, heterogeneous sensor expression, and complexity of architecture in epigenetic sensing in 3D systems compared with 2D cultures [91]. The depth of conventional confocal microscopy is generally limited to the surface of organoids of nominal density (~50–150 m) and, therefore, does not enable real-time imaging of deeper layers. Live-compatible optical clearing strategies have been scaled down to 3D cultures in an attempt to resolve this issue [92]. On-chip clearing methods have enhanced the depth of imaging and maintained cell viability, allowing dynamic observation of intracellular events significantly farther than uncleared samples [86].

The identification of the clearing agent is essential. ClearT2 formulations that include low-molecular-weight polyethylene glycol (PEG) and straightforward high-refractive-index aqueous solutions (e.g., glycerol or iodixanol) have demonstrated consistent enhancements in penetration depth with minor perturbation of live tissues [93]. However, depth-dependent normalization methods are also required since even light clearing may cause variations in refractive index or tissue volume, making the analysis of it quantitatively difficult.

The label-free nonlinear imaging techniques have an advantageous benefit in live organoid studies [81]. Deep structural and metabolic imaging without an exogenous probe is provided by methods, for example, coherent anti-Stokes Raman scattering and second harmonic generation. Still, it is not molecular-specific to epigenetic marks [94]. Consequently, the existing attempts incorporate fluorescent epigenetic reporters into the label-free modalities to an increased degree, where the former defines tissue structure, and the latter describes chromatin movements. Imaging in the near infrared, coupled with excitation by multimodal microscopy, has allowed the fluid and deep penetration of imaging, reducing phototoxicity, allowing long-term imaging of the organoids in regenerative medicine, tumor biology, and drug discovery.

### 4.2. Live Models

Application of epigenetic sensors in vivo is a significant conceptual change whose technical implementation is still challenging. Although real-time imaging of the dynamics of chromatin in living animals is still not feasible, ex vivo imaging and intravital imaging techniques have advanced in a very short time [95]. Light-sheet microscopy with tissue clearing has also made it possible to image whole organs in 3D at depths of millimeters, and this is a hundredfold deeper than the penetration limits of two-photon intravital microscopy [96]. These techniques maintain spatial information and enable one to visualize chromatin structure of whole tissues such as the brain, liver, and kidney [96].

In vivo imaging has been made available through the recent advances in reversible tissue transparency [97]. With food-grade dyes, e.g., tartrazine, modulation of refractive index has made it possible to visualize fluorescently labelled neurons and vasculature in otherwise opaque tissues in live mice without permanently altering the tissue. Even though these methods currently focus on structural and functional imaging, they offer a way forward in the application of GECs in living organisms in the future.

PDX models are an essential step between organoids and clinical practice [98]. Planting organoids into animal models with immunocompromised systems permits time-lapse assessment of the tumor growth, pharmacological reaction, and microenvironmental alteration. Spatially resolved epigenetic heterogeneity in transplanted tumors revealed by integrated imaging and multi-omic profiling has demonstrated the additional utility of live sensing in heterogeneous non-culture in vivo environments.

### 4.3. Influence of Microenvironment

Tissue-specific microenvironments play a strong role in determining the epigenetic state and have a direct impact on sensor performance and interpretability in 3D systems [89]. Against 2D cultures, patient-derived organoids (PDOs) maintain native architecture and cell-cell interactions and, therefore, give more precise responses to epigenetic drugs. Indicatively, triple-negative breast cancer organoids better capture therapy responses, highlighting the importance of 3D organization in changing the role of chromatin regulation [87]. The hypoxia, the stiffness of the extracellular matrix, and the mechanical stress exert strong influences on the histone modification landscapes, as microenvironmental cues [99]. Time-lapse studies of ovarian cancer organoids cultured in collagen matrices have shown dynamic interactions between matrix mechanics and transitions in epigenetic state, and the importance of having sensors resistant to mechanically heterogeneous conditions.

The most significant benefit of organoid systems is that they maintain intra-tumoral heterogeneity [100]. This heterogeneity, however, makes quantitative analysis difficult. The Ratiometric sensor design, which scales the changes at a node to reference channels, has been shown to reduce variability in expression and imaging depth. These techniques in esophageal cancer PDOs allowed the identification of a heterogeneous response to chemoradiotherapy that was close to that of the patients [101]. Taken together, these results make organoids an invaluable platform to test the validity of epigenetic sensors in the physiologically relevant conditions.

### 4.4. Role in Drug Response Analysis

Combining epigenetic sensors and PDOs is transforming drug discovery and personalized medicine. Drug response assays with organoids have been demonstrated to be highly concordant with patient outcomes, and predictive accuracy has been reported to be 80 to 90% in various forms of cancer [102]. PDO assays were found to have a sensitivity of 100% and a specificity of 80% in esophageal cancer to predict the response to chemoradiotherapy [101]. Although these outcomes are encouraging, the majority of research is small-cohort based; there is a need to conduct larger, multi-center confirmations.

Large-scale screening technologies that keep organoids cultured in extracellular matrix during the assay have made possible the systematic screens of extensive compound libraries with no artificial transcriptional alterations [103].

The integration of epigenetic sensors, high-content 3D imaging and machine-learning analysis has offered a mechanistic understanding of drug reactions, such as DNA damage signaling and chromatin remodeling [89]. Nevertheless, computational models are to be viewed with some caution, and the problem of overfitting and low generalizability is still evident.

More sophisticated systems using autologous immune cells and perfusion systems also come closer to a native tumor microenvironment [104]. Longitudinal monitoring of epigenetic evolution in the face of therapeutic pressure can be done using these dynamic models to facilitate research on resistance and immunomodulation.

## 5. Clinical Significance

Live-cell epigenetic sensors provide a distinct chance to connect chromatin biology to clinical decision-making by allowing real-time, functional reporting of epigenetic conditions. Not as substitutes to existing sequencing-based diagnostics, they are used to complement the more traditional static assays, unraveling the temporal dynamics, heterogeneity, and treatment-induced adaptation. Examples of typical clinical uses are listed in Table 4.

### 5.1. Early Disease Detection

There is a tendency for epigenetic changes that precede apparent disease, especially cancer and neurodegeneration [87]. Even though live epigenetic sensors are yet to be directly applied in clinical screening, PDO-based sensor platforms hold a strong discovery engine in detecting early epigenetic signatures. Mapping of these dynamic thresholds in vivo can be used to inform the development of future liquid biopsy markers or targeted imaging probes in the early detection of the disease [108].

Epigenetic dysregulation in neurodegenerative disease is reversible in the early disease stages but is extremely context- and cell-type-specific [109]. Live sensors can be used to identify therapeutic windows in which disease pathways can still be changed by epigenetic interventions, including HDAC inhibition. The same can be said about the metabolic disorders, in which dynamic responses in chromatin to nutrient availability can be monitored in real time.

### 5.2. Treatment Response Monitoring

The main translational benefit of epigenetic sensors is that they can continuously track treatment response in heterogeneous systems [88]. Compared to endpoint assays, live imaging unveils primary adaptive reactions and newly developed resistant subpopulations. Epigenetic vulnerabilities in organoid-based screening are not observed in 2D cultures, which confirms the translational context of these models [99].

Resistance stratification has also been used with live sensing. Indicatively, integrating epigenetic reporters with fluorescence-activated cell sorting has revealed that breast cancer subpopulations with high H3K9me3 are more likely to acquire doxorubicin resistance. Still, those with high levels of DNA methylation marks are more likely to respond to treatment. These insights can be used to have rational combination therapies that can delay resistance.

### 5.3. Opportunities in Regenerative Medicine

Epigenetic sensors are also used in regenerative medicine as real-time quality control instruments in stem cell differentiation [33]. The observation of the global dynamics of H3K9me3 in neuronal differentiation has demonstrated that there is a unique chromatin order reorganization that can be easily tracked and thus promptly identify aberrant epigenetic changes that may undermine therapeutic safety.

The use of epigenetic sensors with PDO biobanks in precision oncology can be used to determine individualized therapeutic testing, synergistic drug combinations, and longitudinal evolution of tumors [110]. Immunotherapy via these platforms is also a prospect, in which epigenetic control of immune checkpoints and tumor immunogenicity is emerging as a new paradigm of response [105].

Although they have promise, there are still considerable hurdles to translation. GECs insertion and especially large dCas9-based constructs have limitations in their delivery due to viral packaging capacity and immunogenicity [111]. The long-term biosafety and possible chromatin perturbation and ethical issues of real-time epigenetic monitoring are to be considered attentively [112]. Regulatory models will have to evolve towards functional diagnostics generating dynamic but not static biomarkers.

## 6. Challenges, Future Directions, and Translational Outlook

### 6.1. Technical Challenges

#### 6.1.1. Delivery and Specificity

An essential challenge is to have high specificity and not to perturb native chromatin dynamics. Numerous reader domains (e.g., chromodomains, zinc-finger motifs) are partially cross-reactive with other similar histone modifications, resulting in the creation of ambiguous signals in the complex 3D environment [113]. Endogenous chromatin reader competition can also cause residence times to change or perturb phase-separated nuclear compartments.

Some of the strategies to enhance specificity are multimerization of the weak-binding domains to take advantage of avidity effects and the application of engineered scFvs that exhibit high specificity with minimal cellular disruption [78]. However, sensor operation has to be tested in physiologically realistic systems in 3D, where crowding, pH gradients, and ionic strength can have a profound impact on binding kinetics [114]. An alternative solution is provided by a cross-reactive sensor array and machine-learning-based pattern recognition, which makes use of controlled promiscuity to develop resistant signatures in heterogeneous systems. Nonetheless, their practical implementation is now limited by low interpretability and batch-to-batch reproducibility [115].

Organoid-based sensor delivery presents other problems, and in vivo. Lentiviruses are examples of viral vectors that can penetrate dense tissues, but have diffusion barriers [99]. Organoid dissociation and reaggregation destroy native architecture but enhance transduction efficiency. Non-integrating methods, for example, mRNA delivery, lessen the danger to the genome while not being effective in intact 3D tissues. Large FRET constructs are also associated with sensor-associated toxicity, which again limits long-term applications, and physiological limits like the blood-brain barrier also make in vivo applications difficult [97].

#### 6.1.2. Multiplex Imaging and Signal Resolution

The epigenetic regulation process is dynamic in nature, necessitating sensors that show high binding kinetics and reversibility. SMT experiments have demonstrated that transient interactions between many chromatin regulators are preferentially revealed by sensor designs with moderate affinity, sensitive enough to detect transient interactions [116].

The photon budget and spectral overlap restrict the measurements of several epigenetic marks at the same time. Other tools, such as FLIM, photoswitchable fluorophores, and chemical-genetic labelling (e.g., SNAP- or HALO-tags), can partially solve this problem [35]. Nevertheless, higher multiplexing is usually associated with a loss of both temporal resolution and long-term compatibility with host cells, resulting in metabolic overhead and the reiteration of exogenous ligands [117].

Background signal and depth-dependent attenuation are also problems with 3D cultures, and complicate quantification. The imaging depth that can be enhanced using optical clearing and refractive index matching may induce physiological perturbations, such as tissue shrinkage and fluorescence preservation, making them less applicable to long-term live imaging applications [118]. To be able to differentiate between biological signals and imaging artifacts, depth-dependent correction and metabolic monitoring are necessary.

### 6.2. Biological and Clinical Translation Challenges

The combination of epigenetics, live-cell imaging, and spatial multi-omics allows for a broader perspective of regulatory conditions. Multicolor immunofluorescence, spatial transcriptomics, and spatial ATAC-seq are endpoint spatial epigenomic profiling technologies that have shown clinically relevant patterns of chromatin accessibility and cell-type-specific regulatory programs in disease settings [85]. Computational models, e.g., MaxFuse, are easy to use in multimodal data integration; however, they are vulnerable to batch effects and platform variability.

Longitudinal multi-omics studies are exceptionally robust in organoid systems, in which epigenetic and transcriptional and phenotypic evolution can be followed simultaneously as they grow and respond to treatments [119]. Nonetheless, clinical translation also has ethical and regulatory issues, including informed consent, patient data privacy, and ownership of patient-derived models [120]. Regulatory programs should also adjust to the functional diagnostics, which produce dynamic, kinetic outputs instead of biomarkers that are not dynamic.

Clinical adoption will depend on standardization based on kinetic benchmarks, reference organoid lines, and multi-center validation trials. Lastly, the equitable access will involve automation, reduction in costs, and distributed screening models to prevent limiting these technologies to special centers [110].

### 6.3. Emerging Opportunities and Future Directions

Recent advances in epigenetic sensor technologies have opened new paths for interrogating dynamic regulatory processes in living systems; however, substantial conceptual and technical challenges remain. A key priority for future development is the expansion of sensing platforms beyond well-characterized histone acylation and methylation marks to encompass emerging epigenetic modifications, including histone acylations such as crotonylation, lactylation, propionylation, and butyrylation, as well as less-explored RNA base modifications. Although these marks are increasingly implicated in metabolic rewiring, inflammatory signaling, and disease progression, their low abundance, rapid enzymatic turnover, and strong dependence on intracellular metabolite availability pose significant obstacles to real-time detection in live cells.

Subsequent research should conceptualize epigenetic regulation as dynamic “epigenetic flux,” as a spatial, temporal, and metabolic component of a 4D (four-dimensional) epigenomics model and not as static marks. Recent research indicates that metabolism is directly connected to chromatin modification states in the form of cofactor availability of acetyl-CoA and SAM, and cellular metabolism connects with epigenetic regulation [121]. Simultaneously, a microenvironmental context in epigenetic responses is emphasized by its role, as evidenced by the spatial heterogeneity of chromatin states in 3D tissues and organoid models [122].

Sensor performance is further limited by the lack of high-affinity, modification-specific reader domains, which constrains both specificity and dynamic range. Addressing these limitations will require coordinated advances in molecular recognition strategies, affinity engineering, and rigorous calibration frameworks capable of capturing transient, metabolically coupled epigenetic states with quantitative accuracy. Minimal-affinity engineered readers, inducible or transient sensor expression systems, and non-competitive nanobody-based recognition are some of the emerging techniques that could help reduce perturbation of endogenous chromatin readers and reduce biological interference during live-cell measurements.

The minimal-affinity engineered reader domains enable temporary interaction with chromatin marks without destabilizing endogenous binding equilibria. Moreover, inducible or transient expression systems are capable of confining sensor activity, removing long-term chromatin perturbation. New technologies also utilize nanobody-based recognition modules, which have smaller binding interfaces and less steric interference than traditional antibody fragments [123]. Such design methods can facilitate epigenetic sensing in live cells that are more physiologically compatible.

An additional frontier lies in integrating live-cell epigenetic sensing with multi-omics approaches. In principle, coupling dynamic imaging of chromatin or RNA modifications with downstream single-cell transcriptomics, chromatin accessibility assays, proteomics, or spatial profiling could establish direct links between epigenetic dynamics and functional cellular outcomes. However, practical implementation is hindered by temporal mismatches between continuous imaging and endpoint sequencing assays, sampling bias, cell loss during downstream processing, and computational uncertainty in aligning dynamic trajectories with static molecular snapshots. While early proof-of-concept studies combining live reporters with single-cell transcriptomics have emerged, most current integrations remain largely inferential rather than mechanistically validated. The future development will rely on experimental designs that will include lineage tracing, synchronous perturbations, and probabilistic computation frameworks that can bridge epigenetic dynamics and cell fate choices and can predictively model epigenetic transition state changes.

Also, live epigenetic sensing can offer insight into higher-order nuclear organization beyond molecular detection. The nuclear mechanical forces and phase-separated chromatin condensates have been increasingly known as controllers of gene expression [124]. Sensors that can record the dynamics of chromatin condensation and metabolite-sensitive epigenetic changes and nuclear mechanics could thus provide insight into the convergence of metabolism, physical forces, and epigenetic regulation in spatially heterogeneous three-dimensional tissues and organoids.

### 6.4. Translational Outlook

From a translational perspective, epigenetic sensors hold promise for informing drug discovery and preclinical evaluation, yet their clinical deployment remains distant. Major barriers include the lack of inter-laboratory standardization, variability in sensor expression and calibration, challenges in interpreting dynamic epigenetic signatures within heterogeneous patient-derived samples, and uncertainty regarding the regulatory classification of live functional assays.

**Figure 4:**
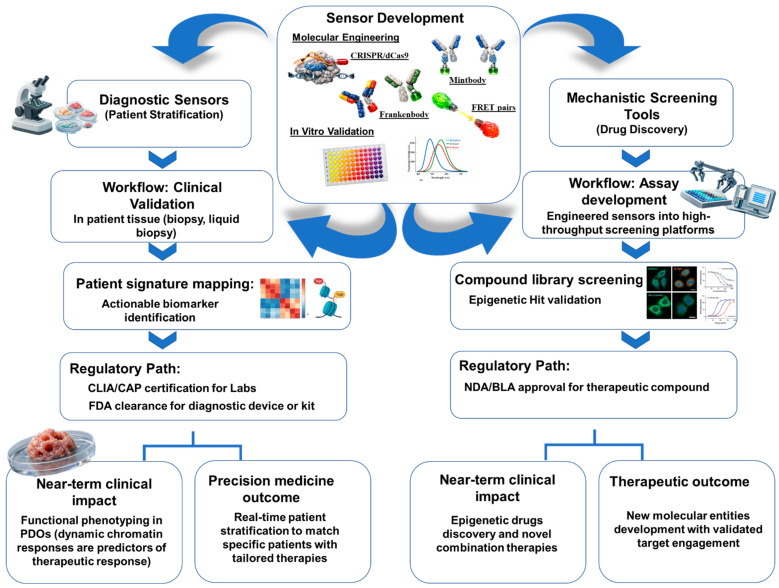
Translation roadmap of epigenetic sensors differentiating corresponding pathways for patient stratification and drug discovery applications.

Notably, translational uses can be split into two mutually complementary directions: diagnostic sensing platforms helpful in real-time patient stratification and mechanistic screening tools useful in high-throughput drug discovery, as shown in Figure 4. Different validation and regulation frameworks govern such applications and must thus have different development strategies.

In the near term, the most feasible applications are likely to arise in patient-derived organoids, ex vivo cultures, and advanced preclinical models, where sensors can be leveraged to monitor chromatin responses to therapeutic perturbations, identify early indicators of drug resistance, and guide the optimization of epigenetic interventions. Potentially, the most effective functional phenotyping of three-dimensional patient-derived organoids is possible, where dynamic chromatin responses may be an early predictor of therapeutic efficacy or resistance before more traditional clinical endpoints.

Such a framework must explicitly account for regulatory, ethical, and logistical constraints, including biosafety considerations associated with genetically encoded constructs, data governance for high-dimensional functional readouts, and the scalability of imaging-based assays in clinical contexts. Overcoming these challenges will require coordinated advances in delivery modalities, imaging infrastructure, computational analytics, and regulatory science.

### 6.5. Conclusions

Although epigenomic profiling technologies have advanced substantially, most remain optimized for static, population-averaged measurements. Sequencing-based approaches provide unmatched molecular specificity and genome-wide coverage; however, their reliance on fixation or cell destruction fundamentally limits the direct interrogation of temporal dynamics, spatial heterogeneity, and microenvironmental regulation.

Live-cell epigenetic sensing approaches, including FRET- and FLIM-based reporters, CRISPR/dCas-derived systems, optogenetic tools, and super-resolution imaging, enable spatially and temporally resolved interrogation of epigenetic states in living cells and three-dimensional models. These methods are uniquely suited to dissect dynamic interactions among histone modifications, RNA marks, chromatin accessibility, and higher-order genome organization within defined nuclear and cellular contexts. Nevertheless, their implementation poses important challenges, including potential perturbation of endogenous chromatin readers, limited multiplexing capacity in living systems, and the difficulty of disentangling global epigenetic trends from locus-specific regulatory events.

Future progress in the field is, therefore, likely to depend less on replacing established epigenomic tools and more on their strategic integration. Priorities include the development of robust calibration frameworks linking sensor outputs to orthogonal biochemical standards, engineering of minimally perturbative sensors, and rational multiplexing strategies that preserve temporal resolution.

In the future, epigenetics is expected to become a predictive and controllable science, rather than a descriptive mapping field. Live epigenetic sensing combined with multi-omics analysis, computational modeling, and programmable technologies of epigenetic editing can eventually make it possible to monitor and manipulate chromatin states in living cells in real-time. In this new paradigm of four-dimensional epigenomics, the quantification of dynamic epigenetic flux across spatially structured tissues will be essential to comprehend the overall interactions of metabolism, nuclear architecture, and chromatin regulation in identifying the cellular identity and disease progression.

## Figures and Tables

**Table 1:** Live and dynamic epigenetic sensors of chromatin modifications.

Sensing Approach	Aim	Principle	Model System	Application	Ref.
Dual FRET biosensor	H3K9me3/H3S10p	Intermolecular FRET	HeLa cells	Anticorrelated mitotic dynamics	[32]
MPP8-Green probe	H3K9me3	Chromodomain-based fluorescent sensor	iPSC differentiation	Global H3K9me3 reorganization	[33]
BiFC epigenetic probe	H3K9me3, 5mC	Split GFP complementation	MCF7 cells	Epigenetic heterogeneity & drug response	[34]
Photostable FRET pairs	Chromatin-binding proteins	Enhanced photostability	Mammalian cells	Long-term single-molecule tracking	[35]
Mintbodies/Fab probes	Histone PTMs	Intracellular antibody fragments	Multiple cell lines	Non-invasive live PTM imaging	[36]
CRISPR-dCas9 imaging	Genomic loci	SgRNA-aptamer recruitment	Mouse ES cells	Real-time locus tracking	[37]
CliF FRET sensor	Protein clustering	Intermolecular FRET	T cells	Proof-of-principle chromatin sensing	[38]
Split GFP systems	Protein interactions	GFP complementation	Various models	Dynamic interaction mapping	[39]
PEG-LANA-DSSMe 11	H2BK120	Synthetic chemical catalyst	Living cells	Modulation of H2BK120 acetylation; suppression of H2B ubiquitination	[40]
Fab-based Probe	H3K27ac, RNAP2-Ser5ph	Endogenous modification labelling	Living cells	Chromatin dynamics	[41]

**Table 2:** Summarized comparison of the above-referenced sensing approaches for epigenetic modifications and their trade-offs.

Live-Cell Compatibility	Sensor Type	Target	Spatial Resolution	Temporal Resolution	Advantages	Limitations
No	Conventional assays (CUT&Tag, ChIP-seq, ATAC-seq, bisulfite sequencing)	DNA methylation, chromatin accessibility, and histone PTMs.	Genome-wide, population-level	Endpoint	High molecular specificity, genomic coverage	need fixation or lysis; ensemble averaging; loss of spatial & temporal dynamics
Live but perturbative	dCas9-based genomic sensors	Locus-specific chromatin state	Single locus	Minutes–hours	Genomic addressability; locus specificity	Steric blocking of transcription; altered chromatin looping; prolonged residence times
	Plasmonic sensors	Nucleosome states, PTMs	Surface confined	Real-time	Highly sensitive, label-free detection	Integration complexity, limited intracellular applicability
	Super-resolution imaging (STORM, PALM)	Chromatin architecture and PTM clusters	<50 nm	Static or slow	Nanoscale spatial information	Phototoxicity; limited temporal resolution
Yes	Mintbodies/intrabodies	Specific histone PTMs (e.g., H3K9ac, H3K27me3)	Nuclear/subnuclear	Seconds–minutes	Live-cell tracking; reversible binding; genetic encodability	Stoichiometric competition with endogenous readers; altered chromatin residence times; potential buffering of PTM dynamics
	FRET-based histone sensors	Intramolecular PTM state	Nanometer-scale (molecular)	Seconds	Quantitative ratiometric output; reversible	Limited locus specificity; linker design constraints; potential steric hindrance
	FLIM-FRET sensors	Histone or protein modification states	Sub-diffraction (lifetime-based)	Seconds–minutes	Concentration-independent quantitation; robust to bleaching	Specialized instrumentation; lower throughput
	Reader-domain-based fusion sensors	Histone PTMs	Locus-level agnostic nuclear	Seconds–minutes	Tunable affinity, modular strategy	Chromatin decompaction artifacts at high expression; cross-reactivity among PTMs; prolonged occupancy
	Multiplexed Dark FRET	Multiple PTMs simultaneously	Molecular	Seconds	Multiplexing capability; reduced spectral overlap	Complex calibration; signal deconvolution challenges
	Cas13-based RNA modification sensors	RNA abundance or modification	Single RNA species	Seconds–minutes	Direct RNA targeting; dynamic readout	Off-target RNA binding; limited modification specificity
	Microfluidic/organ-on-chip platforms	Integrated epigenetic responses	Tissue-level	Minutes–hours	Controlled microenvironment; drug screening	Optical penetration limits; delivery challenges
	Aptamer/RNA-based sensors	Histone PTMs, metabolites, RNAs	Molecular	Seconds	Small size; minimal steric hindrance	Stability issues; intracellular delivery challenges

**Table 3:** Applications of epigenetic sensing and spatial epigenomic profiling approaches in 3D culture and organoid models.

Approach	Disease/Condition	Model Type	Findings	Ref.
Fab-based H3K27ac/H3K9ac live-cell imaging	Embryogenesis	Whole embryo (somatic cell nuclear transfer)	Dynamics of histone acetylation during development	[84]
CODEX multiplex immunofluorescence + spatial chromatin profiling	Angioimmunoblastic T-cell lymphoma	Patient FFPE tissue sections	Spatial ATAC-seq and DBiT-seq revealed high chromatin accessibility	[85]
Optical clearing +H3K9me3 immunofluorescence	Glioblastoma	Brain organoids	Spatial heterogeneity of heterochromatin	[86]
Organoid-based epigenetic drug screening	Breast cancer	PDOs	Identification of epigenetic drug hits	[87]
ɤH2AX imaging	Ovarian cancer	PDOs	Dose-dependent DNA damage responses	[88]
Live epigenetic imaging	Pediatric brain tumors	Organoids/xenografts	SUV39H inhibition sensitivity	[89]
Spatial ATAC-seq + RNA-seq multi-omics	Pancreatic cancer	PDOs	Chromatin accessibility drug response associations	[90]

**Table 4:** Clinical and translational applications of epigenetic sensor technologies.

Disease Area	Epigenetic Target	Sensor Platform	Clinical Application	Ref.
Ovarian cancer	DNA damage response (H2AX)	High-content imaging organoids	Chemotherapy response prediction; resistant model identification	[88]
Pediatric brain tumors	SUV39H (H3K9 methylation)	Tumor organoids from xenografts	High throughput epigenetic drug screening ± radiation	[89]
Neuronal differentiation	H3K9me3 dynamics	MPP8-Green probe	Quality control in iPSC differentiation	[33]
Pan-cancer immunotherapy	Multiple immune + epigenetic markers	Organoid-immune cell co-culture	Functional testing of immunotherapies; immune infiltration monitoring	[105]
Pancreatic cancer	KMT2C, various histone modifications	3D CRC organoids with multi-omic profiling	Drug screening, correlation of epigenetic state with treatment response	[99]
High-grade, serious ovarian cancer	Multiple epigenetic pathways	PDOs with RNA-seq	Disease modeling; evolutionary characterization; drug screening	[106]
Endometrial cancer	MMR, p53, POLE mutations	Molecular subtyping	Subtype-specific drug screening; personalized treatment	[107]
Bladder cancer	Chromatin accessibility, gene expression	Multi-omic profiling	Ex vivo drug response prediction; biomarker identification for gemcitabine	[100]
Neuroendocrine cervical cancer (rare)	Various epigenetic modifications	Organoid-based automated HTS platform	Rapid drug screening for rare malignancies with unmet needs	[103]

## Data Availability

No new data were created or analyzed in this study. Data sharing is not applicable to this article.

## Abbreviations

The following abbreviations are used in this manuscript:

3D	Three-Dimensional
ATAC-seq	Assay of Transposase-Accessible Chromatin Sequencing
BiFC	Bimolecular Fluorescence Complementation
ChIP	Chromatin Immunoprecipitation
CRC	Colorectal Cancer
CRISPR	Clustered Regularly Interspaced Short Palindromic Repeats
DNA	Deoxyribonucleic Acid
DNMT	DNA Methyltransferase
FLIM	Fluorescence Lifetime Imaging
FP	Fluorescent proteins
FRET	Förster Resonance Energy Transfer
GECs	Genetically Engineered Sensors
HDACs	Histone Deacetylases
HDMS	Histone Demethylases
iPSC	Induced Pluripotent Stem Cell
Live-MIEL	Live Cell Microscopic Imaging of Epigenetic Landscapes
LSPR	Localized Surface Plasmon Resonance
m6A	N6-methyladenosine
MDF	Multiplexed Dark FRET
mintbodies	Modification-Specific Intracellular Antibodies
mRNA	Messenger Ribonucleic Acid
PDO	Patient-Derived Organoid
PEG	Polyethylene Glycol
RNA	Ribonucleic Acid
ROS	Reactive Oxygen Species
SAM	S-adenosylmethionine
scFv	Single-Chain Variable Fragments
SHERLOCK	Specific High-Sensitivity Enzymatic Reporter Unlocking
SMT	Single-Molecule Tracking
STED	Stimulated Emission Depletion
STEDD	Stimulated Emission Double Depletion

## References

[1] Lungu C., Pinter S., Broche J., Rathert P., Jeltsch A. (2017). Modular fluorescence complementation sensors for live cell detection of epigenetic signals at endogenous genomic sites. Nat. Commun..

[2] Wu Y.-L., Lin Z.-J., Li C.-C., Lin X., Shan S.-K., Guo B., Zheng M.-H., Li F., Yuan L.-Q., Li Z.-h. (2023). Epigenetic regulation in metabolic diseases: Mechanisms and advances in clinical study. Signal Transduct. Target. Ther..

[3] Dai W., Qiao X., Fang Y., Guo R., Bai P., Liu S., Li T., Jiang Y., Wei S., Na Z. (2024). Epigenetics-targeted drugs: Current paradigms and future challenges. Signal Transduct. Target. Ther..

[4] Chen X., Xu H., Shu X., Song C.-X. (2025). Mapping epigenetic modifications by sequencing technologies. Cell Death Differ..

[5] Khan S., Shukla S., Sinha S., Meeran S.M. (2016). Epigenetic targets in cancer and aging: Dietary and therapeutic interventions. Expert Opin. Ther. Targets.

[6] Grandi F.C., Modi H., Kampman L., Corces M.R. (2022). Chromatin accessibility profiling by ATAC-seq. Nat. Protoc..

[7] Chu L.-X., Wang W.-J., Gu X.-P., Wu P., Gao C., Zhang Q., Wu J., Jiang D.-W., Huang J.-Q., Ying X.-W. (2024). Spatiotemporal multi-omics: Exploring molecular landscapes in aging and regenerative medicine. Mil. Med. Res..

[8] Tosat-Bitrian C. (2022). Biosensors to Evaluate Drug Efficacy in Neurodegenerative Diseases. https://docta.ucm.es/entities/publication/035bee1e-cd76-4001-86e6-48c665c3ca95.

[9] Mehrmohamadi M., Sepehri M.H., Nazer N., Norouzi M.R. (2021). A Comparative Overview of Epigenomic Profiling Methods. Front. Cell Dev. Biol..

[10] Sánchez O.F., Mendonca A., Min A., Liu J., Yuan C. (2019). Monitoring Histone Methylation (H3K9me3) Changes in Live Cells. ACS Omega.

[11] Liu W., Cui Y., Ren W., Irudayaraj J. (2019). Epigenetic biomarker screening by FLIM-FRET for combination therapy in ER+ breast cancer. Clin. Epigenetics.

[12] Tang H., Peng J., Jiang X., Peng S., Wang F., Weng X., Zhou X. (2023). A CRISPR-Cas and Tat Peptide with Fluorescent RNA Aptamer System for Signal Amplification in RNA Imaging. Biosensors.

[13] Xu X., Qi L.S. (2019). A CRISPR–dCas Toolbox for Genetic Engineering and Synthetic Biology. J. Mol. Biol..

[14] Dimitrova E., Turberfield A.H., Klose R.J. (2015). Histone demethylases in chromatin biology and beyond. EMBO Rep..

[15] Lennartsson A., Ekwall K. (2009). Histone modification patterns and epigenetic codes. Biochim. Biophys. Acta (BBA)-Gen. Subj..

[16] Meas R., Mao P. (2015). Histone ubiquitylation and its roles in transcription and DNA damage response. DNA Repair..

[17] Johansen K.M., Johansen J. (2006). Regulation of chromatin structure by histone H3S10 phosphorylation. Chromosome Res..

[18] Talbert P.B., Henikoff S. (2021). Histone variants at a glance. J. Cell Sci..

[19] Cao G., Li H.-B., Yin Z., Flavell R.A. (2016). Recent advances in dynamic m6A RNA modification. Open Biol..

[20] Artika I., Made R., Arianti M.Á., Demény E.K. (2025). RNA modifications and their role in gene expression. Front. Mol. Biosci..

[21] Flamand M.N., Tegowski M., Meyer K.D. (2023). The Proteins of mRNA Modification: Writers, Readers, and Erasers. Annu. Rev. Biochem..

[22] Chen B., Yuan B.-F., Feng Y.-Q. (2018). Analytical methods for deciphering RNA modifications. Anal. Chem..

[23] Corces M.R., Granja J.M., Shams S., Louie B.H., Seoane J.A., Zhou W., Silva T.C., Groeneveld C.W., Christopher K., Cho S.W. (2018). The chromatin accessibility landscape of primary human cancers. Science.

[24] Zhao Y., Dong Y., Hong W., Jiang C., Yao K., Cheng C. (2022). Computational modeling of chromatin accessibility identified important epigenomic regulators. BMC Genom..

[25] Fischle W., Wang Y., Allis C.D. (2003). Histone and chromatin cross-talk. Curr. Opin. Cell Biol..

[26] Farkas G., Leibovitch B.A., Elgin S.C.R. (2000). Chromatin organization and transcriptional control of gene expression in Drosophila. Gene.

[27] Liu J., Liu L., He J., Xu Y., Wang Y. (2021). Multi-omic analysis of altered transcriptome and epigenetic signatures in the UV-induced DNA damage response. DNA Repair..

[28] Caicedo J.C., Cooper S., Heigwer F., Warchal S., Qiu P., Molnar C., Vasilevich A.S., Barry J.D., Bansal H.S., Kraus O. (2017). Data-analysis strategies for image-based cell profiling. Nat. Methods.

[29] Mirny L.A., Imakaev M., Abdennur N. (2019). Two major mechanisms of chromosome organization. Curr. Opin. Cell Biol..

[30] Pal K., Forcato M., Ferrari F. (2019). Hi-C analysis: From data generation to integration. Biophys. Rev..

[31] Deng W., Shi X., Tjian R., Lionnet T., Singer R.H. (2015). CASFISH: CRISPR/Cas9-mediated in situ labeling of genomic loci in fixed cells. Proc. Natl. Acad. Sci. USA.

[32] Peng Q., Lu S., Shi Y., Pan Y., Limsakul P., Chernov A.V., Qiu J., Chai X., Shi Y., Wang P. (2018). Coordinated histone modifications and chromatin reorganization in a single cell revealed by FRET biosensors. Proc. Natl. Acad. Sci. USA.

[33] Stepanov A.I., Shuvaeva A.A., Putlyaeva L.V., Lukyanov D.K., Galiakberova A.A., Gorbachev D.A., Maltsev D.I., Pronina V., Dylov D.V., Terskikh A.V. (2024). Tracking induced pluripotent stem cell differentiation with a fluorescent genetically encoded epigenetic probe. Cell. Mol. Life Sci..

[34] Mendonca A., Sánchez O., Zhao H., Lin L., Min A., Yuan C. (2022). Development and application of novel BiFC probes for cell sorting based on epigenetic modification. Cytom. Part A.

[35] Basu S., Needham L.-M., Lando D., Taylor E.J., Wohlfahrt K.J., Shah D., Boucher W., Tan Y.L., Bates L.E., Tkachenko O. (2018). FRET-enhanced photostability allows improved single-molecule tracking of proteins and protein complexes in live mammalian cells. Nat. Commun..

[36] Sato Y., Kujirai T., Arai R., Asakawa H., Ohtsuki C., Horikoshi N., Yamagata K., Ueda J., Nagase T., Haraguchi T. (2016). A Genetically Encoded Probe for Live-Cell Imaging of H4K20 Monomethylation. J. Mol. Biol..

[37] Fu Y., Rocha P.P., Luo V.M., Raviram R., Deng Y., Mazzoni E.O., Skok J.A. (2016). CRISPR-dCas9 and sgRNA scaffolds enable dual-colour live imaging of satellite sequences and repeat-enriched individual loci. Nat. Commun..

[38] Ma Y., Pandzic E., Nicovich P.R., Yamamoto Y., Kwiatek J., Pageon S.V., Benda A., Rossy J., Gaus K. (2017). An intermolecular FRET sensor detects the dynamics of T cell receptor clustering. Nat. Commun..

[39] Delehanty J.B., Medintz I.L. (2012). Elaborate Nanoparticle-Based Traps for Catching Cytosolic Players in the Act. ChemBioChem.

[40] Fujiwara Y., Yamanashi Y., Fujimura A., Sato Y., Kujirai T., Kurumizaka H., Kimura H., Yamatsugu K., Kawashima S.A., Kanai M. (2021). Live-cell epigenome manipulation by synthetic histone acetylation catalyst system. Proc. Natl. Acad. Sci. USA.

[41] Saxton M.N., Morisaki T., Krapf D., Kimura H., Stasevich T.J. (2023). Live-cell imaging uncovers the relationship between histone acetylation, transcription initiation, and nucleosome mobility. Sci. Adv..

[42] Chung C.-I., Sato Y., Ohmuro-Matsuyama Y., Machida S., Kurumizaka H., Kimura H., Ueda H. (2019). Intrabody-based FRET probe to visualize endogenous histone acetylation. Sci. Rep..

[43] Sato Y., Mukai M., Ueda J., Muraki M., Stasevich T.J., Horikoshi N., Kujirai T., Kita H., Kimura T., Hira S. (2013). Genetically encoded system to track histone modification in vivo. Sci. Rep..

[44] Bertolino M. (2024). Development of a Tool for Tracking H3K27 Tri-Methylation. Master’s Thesis.

[45] Zhao N., Kamijo K., Fox P.D., Oda H., Morisaki T., Sato Y., Kimura H., Stasevich T.J. (2019). A genetically encoded probe for imaging nascent and mature HA-tagged proteins in vivo. Nat. Commun..

[46] Pedelacq J.-D., Cabantous S. (2019). Development and Applications of Superfolder and Split Fluorescent Protein Detection Systems in Biology. Int. J. Mol. Sci..

[47] Thuma J., Chung Y.-C., Tu L.-C. (2023). Advances and challenges in CRISPR-based real-time imaging of dynamic genome organization. Front. Mol. Biosci..

[48] Stepanov A.I., Putlyaeva L.V., Besedovskaya Z., Shuvaeva A.A., Karpenko N.V., Rukh S., Gorbachev D.A., Malyshevskaia K.K., Terskikh A.V., Lukyanov K.A. (2024). Genetically encoded epigenetic sensors for visualization of H3K9me3, H3K9ac and H3K4me1 histone modifications in living cells. Biochem. Biophys. Res. Commun..

[49] Besedovskaia Z., Putlyaeva L., Lukyanov K. LiveMIEL: Genetically encoded epigenetic probes for enhancers dynamics visualization in live-cell fluorescence microscopy. Proceedings of the IECBM 2022.

[50] Hu J.-J., Yang J., Liu Y., Lu G., Zhao Z., Xia F., Lou X. (2024). Tuning the affinity of probes with transmembrane proteins by constructing peptide-conjugated cis/trans isomers based on molecular scaffolds. J. Mater. Chem. B.

[51] Farhy C., Hariharan S., Ylanko J., Orozco L., Zeng F.-Y., Pass I., Ugarte F., Forsberg E.C., Huang C.-T., Andrews D.W. (2019). Improving drug discovery using image-based multiparametric analysis of the epigenetic landscape. Elife.

[52] Khorshid M., Ahi E.P. (2025). RNA Aptamers and Epitranscriptomics: Charting Unexplored Territories in RNA Biology. Molecular Diagnosis & Therapy.

[53] Zhang T., Yang H., Yu Q., Zhang Y., Zhang Y., Zhu X., Xia X., Li F., Deng R. (2025). Dynamic, Single-cell Monitoring of RNA Modifications Response to Viral Infection Using a Genetically Encoded Live-cell RNA Methylation Sensor. Angew. Chem. Int. Ed..

[54] Chen Z., Chen W., Reheman Z., Jiang H., Wu J., Li X. (2023). Genetically encoded RNA-based sensors with Pepper fluorogenic aptamer. Nucleic Acids Res..

[55] Peng Q., Cheng B., Lu S., Chien S., Wang Y. (2016). Perspectives of FRET imaging to study epigenetics and mechanobiology in the nucleus. Molecular And Cellular Mechanobiology.

[56] Gong Y., Wei C., Cheng L., Ma F., Lu S., Peng Q., Liu L., Wang Y. (2021). Tracking the Dynamic Histone Methylation of H3K27 in Live Cancer Cells. ACS Sens..

[57] Han Q., Chen F., Liu S., Ge Y., Wu J., Liu D. (2021). Genetically encoded FRET fluorescent sensor designed for detecting MOF histone acetyltransferase activity in vitro and in living cells. Anal. Bioanal. Chem..

[58] Lou J., Scipioni L., Wright B.K., Bartolec T.K., Zhang J., Masamsetti V.P., Gaus K., Gratton E., Cesare A.J., Hinde E. (2019). Phasor histone FLIM-FRET microscopy quantifies spatiotemporal rearrangement of chromatin architecture during the DNA damage response. Proc. Natl. Acad. Sci. USA.

[59] Braun A., Liao E., Vunnam N., Murray M., Sachs J. (2026). Multiplexed Dark FRET Biosensors: An accessible live-cell platform for target- and cell-specific monitoring of protein-protein interactions in 2D and 3D model systems. ACS Sens..

[60] Murakoshi H., Shibata A.C.E. (2017). ShadowY: A dark yellow fluorescent protein for FLIM-based FRET measurement. Sci. Rep..

[61] Chen B., Gilbert L.A., Cimini B.A., Schnitzbauer J., Zhang W., Li G.-W., Park J., Blackburn E.H., Weissman J.S., Qi L.S. (2013). Dynamic Imaging of Genomic Loci in Living Human Cells by an Optimized CRISPR/Cas System. Cell.

[62] Versosky T.J., Nishonov D.U., Tu L.C. (2025). Real-Time Imaging of Specific Genomic Loci With CRISPR/dCas9 in Human Cells Using CRISPRainbow. Bio-Protocol.

[63] Ma H., Tu L.-C., Naseri A., Huisman M., Zhang S., Grunwald D., Pederson T. (2016). CRISPR-Cas9 nuclear dynamics and target recognition in living cells. J. Cell Biol..

[64] Ma H., Naseri A., Reyes-Gutierrez P., Wolfe S.A., Zhang S., Pederson T. (2015). Multicolor CRISPR labeling of chromosomal loci in human cells. Proc. Natl. Acad. Sci. USA.

[65] Wang H., Nakamura M., Abbott T.R., Zhao D., Luo K., Yu C., Nguyen C.M., Lo A., Daley T.P., La Russa M. (2019). CRISPR-mediated live imaging of genome editing and transcription. Science.

[66] Zhang K., Deng R., Teng X., Li Y., Sun Y., Ren X., Li J. (2018). Direct visualization of single-nucleotide variation in mtDNA using a CRISPR/Cas9-mediated proximity ligation assay. J. Am. Chem. Soc..

[67] Kellner M.J., Koob J.G., Gootenberg J.S., Abudayyeh O.O., Zhang F. (2019). SHERLOCK: Nucleic acid detection with CRISPR nucleases. Nat. Protoc..

[68] Tam J., Merino D. (2015). Stochastic optical reconstruction microscopy (STORM) in comparison with stimulated emission depletion (STED) and other imaging methods. J. Neurochem..

[69] Zhang W., Noa A., Nienhaus K., Hilbert L., Nienhaus G.U. (2019). Super-resolution imaging of densely packed DNA in nuclei of zebrafish embryos using stimulated emission double depletion microscopy. J. Phys. D Appl. Phys..

[70] Rhodes A.D.Y., Duran-Mota J.A., Oliva N. (2022). Current progress in bionanomaterials to modulate the epigenome. Biomater. Sci..

[71] Soha S.A., Santhireswaran A., Huq S., Casimir-Powell J., Jenkins N., Hodgson G.K., Sugiyama M., Antonescu C.N., Impellizzeri S., Botelho R.J. (2023). Improved imaging and preservation of lysosome dynamics using silver nanoparticle-enhanced fluorescence. Mol. Biol. Cell.

[72] An H.J., Kim Y., Chang S., Kim H., Song J., Park H., Choi I. (2021). High-spatial and colourimetric imaging of histone modifications in single senescent cells using plasmonic nanoprobes. Nat. Commun..

[73] Xiong Y., Li M., Cao Y., Li Z., Chang Y., Zhao X., Qing G. (2023). Nanofluidic Device for Detection of Lysine Methylpeptides and Sensing of Lysine Methylation. Anal. Chem..

[74] Thenuwara G., Javed B., Singh B., Tian F. (2024). Biosensor-enhanced organ-on-a-chip models for investigating glioblastoma tumor microenvironment dynamics. Sensors.

[75] Emiliani V., Entcheva E., Hedrich R., Hegemann P., Konrad K.R., Lüscher C., Mahn M., Pan Z.-H., Sims R.R., Vierock J. (2022). Optogenetics for light control of biological systems. Nat. Rev. Methods Primers.

[76] Stepanov A.I., Zhurlova P.A., Shuvaeva A.A., Sokolinskaya E.L., Gurskaya N.G., Lukyanov K.A., Putlyaeva L.V. (2023). Optogenetics for sensors: On-demand fluorescent labeling of histone epigenetics. Biochem. Biophys. Res. Commun..

[77] Peng Q., Weng K., Li S., Xu R., Wang Y., Wu Y. (2021). A Perspective of Epigenetic Regulation in Radiotherapy. Front. Cell Dev. Biol..

[78] Sato Y., Nakao M., Kimura H. (2021). Live-cell imaging probes to track chromatin modification dynamics. Microscopy.

[79] Ohmuro-Matsuyama Y., Kitaguchi T., Kimura H., Ueda H. (2021). Simple Fluorogenic Cellular Assay for Histone Deacetylase Inhibitors Based on Split-Yellow Fluorescent Protein and Intrabodies. ACS Omega.

[80] Ashour A.A., Tayeb F.J., Felemban M.F., Shafie A. (2025). Nanomaterial-based biosensors for cancer diagnosis: Trends and innovations. Mikrochim. Acta.

[81] Zhao W., Sun D., Yue S. (2023). Label-free multimodal non-linear optical imaging of three-dimensional cell cultures. Front. Phys..

[82] Farhang Doost N., Srivastava S.K. (2024). A Comprehensive Review of Organ-on-a-Chip Technology and Its Applications. Biosensors.

[83] Vazquez-Armendariz A.I., Tata P.R. (2023). Recent advances in lung organoid development and applications in disease modeling. J. Clin. Investig..

[84] Hayashi-Takanaka Y., Yamagata K., Wakayama T., Stasevich T.J., Kainuma T., Tsurimoto T., Tachibana M., Shinkai Y., Kurumizaka H., Nozaki N. (2011). Tracking epigenetic histone modifications in single cells using Fab-based live endogenous modification labeling. Nucleic Acids Res..

[85] Enninful A., Foss F.M., Fan R., Xu M. (2024). Spatial Multiomics Profiling of Angioimmunoblastic T-Cell Lymphoma. Blood.

[86] Wei M., Shi L., Shen Y., Zhao Z., Guzman A., Kaufman L.J., Wei L., Min W. (2019). Volumetric chemical imaging by clearing-enhanced stimulated Raman scattering microscopy. Proc. Natl. Acad. Sci. USA.

[87] Rao X., Qiao Z., Yang Y., Deng Y., Zhang Z., Yu X., Guo X. (2024). Unveiling epigenetic vulnerabilities in triple-negative breast cancer through 3D organoid drug screening. Pharmaceuticals.

[88] Keles H., Schofield C.A., Rannikmae H., Edwards E.E., Mohamet L. (2023). A Scalable 3D High-Content Imaging Protocol for Measuring a Drug Induced DNA Damage Response Using Immunofluorescent Subnuclear γH2AX Spots in Patient Derived Ovarian Cancer Organoids. ACS Pharmacol. Transl. Sci..

[89] Bae G., Palacios M.S., Powell R.T., Stephan C.C., Li X., Du Y. (2024). Tumor organoid predicted therapeutic responses to novel epigenetic drugs via high-throughput screening. Neuro-Oncology.

[90] Contreras C.T., Schneider G., Müller D., Mirzakhani K., Conrads K., Kaulfuß S., Beißbarth T., Salinas G., Beyer N., Reinländer S. (2024). Patient-Derived Organoid Cultures for Personalized Therapies and Targeted Drug Screening Applications. Cancer Res..

[91] Costa E.C., Silva D.N., Moreira A.F., Correia I.J. (2019). Optical clearing methods: An overview of the techniques used for the imaging of 3D spheroids. Biotechnol. Bioeng..

[92] Kostrikov S., Johnsen K.B., Braunstein T.H., Gudbergsson J.M., Fliedner F.P., Obara E.A., Hamerlik P., Hansen A.E., Kjaer A., Hempel C. (2021). Optical tissue clearing and machine learning can precisely characterize extravasation and blood vessel architecture in brain tumors. Commun. Biol..

[93] Inagaki T., Kim J., Tomida K., Maeda E., Matsumoto T. (2023). 3D quantitative assessment for nuclear morphology in osteocytic spheroid with optical clearing technique. Integr. Biol..

[94] Mazumder N., Balla N.K., Zhuo G.-Y., Kistenev Y.V., Kumar R., Kao F.-J., Brasselet S., Nikolaev V.V., Krivova N.A. (2019). Label-Free Non-linear Multimodal Optical Microscopy—Basics, Development, and Applications. Front. Phys..

[95] Lagerweij T., Dusoswa S.A., Negrean A., Hendrikx E.M., de Vries H.E., Kole J., Garcia-Vallejo J.J., Mansvelder H.D., Vandertop W.P., Noske D.P. (2017). Optical clearing and fluorescence deep-tissue imaging for 3D quantitative analysis of the brain tumor microenvironment. Angiogenesis.

[96] Ueda H.R., Ertürk A., Chung K., Gradinaru V., Chédotal A., Tomancak P., Keller P.J. (2020). Tissue clearing and its applications in neuroscience. Nat. Rev. Neurosci..

[97] Ou Z., Duh Y.-S., Rommelfanger N.J., Keck C.H., Jiang S., Brinson K., Zhao S., Schmidt E.L., Wu X., Yang F. (2024). Achieving optical transparency in live animals with absorbing molecules. Science.

[98] Magill S.T., Vasudevan H.N., Seo K., Villanueva-Meyer J.E., Choudhury A., John Liu S., Pekmezci M., Findakly S., Hilz S., Lastella S. (2020). Multiplatform genomic profiling and magnetic resonance imaging identify mechanisms underlying intratumor heterogeneity in meningioma. Nat. Commun..

[99] Kim J.S., Park C.H., Kim E., Lee H.S., Lee J., Kim J., Kam E.H., Nam S., Chung M.J., Park J.Y. (2025). Establishing 3D organoid models from patient-derived conditionally reprogrammed cells to bridge preclinical and clinical insights in pancreatic cancer. Mol. Cancer.

[100] Merrill N.M., Kaffenberger S.D., Bao L., Vandecan N., Goo L.E., Apfel A., Cheng X., Qin Z., Liu C.-J., Bankhead A. (2024). Abstract A009: Integrative drug screening and multi-omic characterization of patient-derived bladder cancer organoids reveals novel molecular correlates of chemotherapy response. Clin. Cancer Res..

[101] Karakasheva T.A., Gabre J.T., Sachdeva U.M., Cruz-Acuña R., Lin E.W., DeMarshall M., Falk G.W., Ginsberg G.G., Yang Z., Kim M.M. (2021). Patient-derived organoids as a platform for modeling a patient’s response to chemoradiotherapy in esophageal cancer. Sci. Rep..

[102] Vlachogiannis G., Hedayat S., Vatsiou A., Jamin Y., Fernández-Mateos J., Khan K., Lampis A., Eason K., Huntingford I., Burke R. (2018). Patient-derived organoids model treatment response of metastatic gastrointestinal cancers. Science.

[103] Xu Z., Yang H., Zhou Y., Dzakah E.E., Zhao B. (2025). Whole-Process 3D ECM-Encapsulated Organoid-Based Automated High-Throughput Screening Platform Accelerates Drug Discovery for Rare Diseases. Life Med..

[104] Millard M., Dance N., DesRochers T.M. (2023). Perfused patient-derived tumor organoid models with autologous immune cells for preclinical drug development. Cancer Res..

[105] de Man S., Chavez-Abiega S., Veenendaal T., Kop M., Hanrath J., Okkes D., Spanjaard E., Price L., Goverse G. (2024). Abstract B040: High-Content Screening to Enhance the Preclinical Development of Immunotherapies, Validated for 100 Patient-Derived Organoids. Cancer Immunol. Res..

[106] Kallunki T., Lauridsen A.R., Skorda A., Lahtinen A., Bay M.L., Rasmussen B., Huhtinen K., Muranen T., Oikkonen J., Hynninen J. (2024). Establishment and analysis of patient-derived high-grade serous ovarian cancer organoids for disease modeling and drug testing. Cancer Res..

[107] Liu D., Powell E., Wan K.M., Athavale R., Loo C., Ford C.E. (2024). Abstract B014: Patient-derived organoid models for drug screening in molecular subtypes of endometrial cancer. Clin. Cancer Res..

[108] Kelsey G., Stegle O. (2017). Single-cell epigenomics: Recording the past and predicting the future. Science.

[109] Wang R., Wang M., Du D., Shan Z., Bi L., Chen Q.-H. (2025). Brain-Targeted Reactive Oxygen Species in Hypertension: Unveiling Subcellular Dynamics, Immune Cross-Talk, and Novel Therapeutic Pathways. Antioxidants.

[110] Şekeroğlu Z.A., Şekeroğlu V. (2025). A review on patient-derived 3D micro cancer approach for drug screen in personalized cancer medicine. Curr. Cancer Drug Targets.

[111] Mitchell M.J., Billingsley M.M., Haley R.M., Wechsler M.E., Peppas N.A., Langer R. (2021). Engineering precision nanoparticles for drug delivery. Nat. Rev. Drug Discov..

[112] Vayena E., Blasimme A. (2018). Machine learning in medicine: Addressing ethical challenges. PLoS Med..

[113] Gibson B.A., Doolittle L.K., Schneider M.W.G., Jensen L.E., Gamarra N., Henry L., Gerlich D.W., Redding S., Rosen M.K. (2019). Organization of Chromatin by Intrinsic and Regulated Phase Separation. Cell.

[114] Peveler W.J., Algar W.R. (2018). More than a light switch: Engineering unconventional fluorescent configurations for biological sensing. ACS Chem. Biol..

[115] Fitzgerald J.E., Fenniri H. (2016). Biomimetic Cross-Reactive Sensor Arrays: Prospects in Biodiagnostics. RSC Adv..

[116] Huseyin M.K., Klose R.J. (2021). Live-cell single particle tracking of PRC1 reveals a highly dynamic system with low target site occupancy. Nat. Commun..

[117] Kannan S., Peng C.-C., Wu H.-M., Tung Y.-C. (2024). Characterization of single-spheroid oxygen consumption using a microfluidic platform and fluorescence lifetime imaging microscopy. Biosensors.

[118] Nürnberg E., Vitacolonna M., Klicks J., Von Molitor E., Cesetti T., Keller F., Bruch R., Ertongur-Fauth T., Riedel K., Scholz P. (2020). Routine optical clearing of 3D-cell cultures: Simplicity forward. Front. Mol. Biosci..

[119] Gunnarsson E.B., Kim S., Choi B., Schmid J.K., Kaura K., Lenz H.-J., Mumenthaler S.M., Foo J. (2024). Understanding patient-derived tumor organoid growth through an integrated imaging and mathematical modeling framework. PLoS Comput. Biol..

[120] Ren X., Chen W., Yang Q., Li X., Xu L. (2022). Patient-derived cancer organoids for drug screening: Basic technology and clinical application. J. Gastroenterol. Hepatol..

[121] Gao T., Díaz-Hirashi Z., Verdeguer F. (2018). Metabolic Signaling into Chromatin Modifications in the Regulation of Gene Expression. Int. J. Mol. Sci..

[122] Deng Y., Bartosovic M., Ma S., Zhang D., Kukanja P., Xiao Y., Su G., Liu Y., Qin X., Rosoklija G.B. (2022). Spatial profiling of chromatin accessibility in mouse and human tissues. Nature.

[123] De Beer M.A., Giepmans B.N. (2020). Nanobody-based probes for subcellular protein identification and visualization. Front. Cell. Neurosci..

[124] Li X., An Z., Zhang W., Li F. (2023). Phase Separation: Direct and Indirect Driving Force for High-Order Chromatin Organization. Genes.

